# The maternal hair metabolome is capable of discriminating intrahepatic cholestasis of pregnancy from uncomplicated pregnancy

**DOI:** 10.3389/fendo.2023.1280833

**Published:** 2024-01-08

**Authors:** Nanlin Yin, Xiuping Jiang, Muhua Yu, Yang Yang, Huisheng Ge, Ting-Li Han, Hongbo Qi

**Affiliations:** ^1^ Department of Obstetrics and Gynecology, Women and Children’s Hospital of Chongqing Medical University, Chongqing, China; ^2^ Center for Reproductive Medicine, The First Affiliated Hospital of Chongqing Medical University, Chongqing, China; ^3^ Department of Obstetrics and Gynecology, The Second Affiliated Hospital of Chongqing Medical University, Chongqing, China; ^4^ Department of Obstetrics, The First Affiliated Hospital of Chongqing Medical University, Chongqing, China; ^5^ Joint International Research Laboratory of Reproduction and Development of Chinese Ministry of Education, Chongqing Medical University, Chongqing, China; ^6^ Chongqing Key Laboratory of Maternal and Fetal Medicine, Chongqing Medical University, Chongqing, China

**Keywords:** cholestasis of pregnancy, metabolomics, gas chromatography-mass spectrometry, hair, plasma

## Abstract

**Introduction:**

Intrahepatic cholestasis of pregnancy (ICP) is a pregnancy-specific liver disease associated with elevated bile acids in the blood. Diagnosis typically only occurs after the manifestation of clinical symptoms and the metabolic mechanisms underlying its development remain unclear. The aim of this study was to investigate potential specific metabolites and the underlying metabolic changes occurring during the development of ICP in the maternal plasma and hair metabolomes of women diagnosed with either ICP or having a healthy pregnancy.

**Methods:**

A total of 35 Chinese women with ICP and 42 healthy pregnancies were enrolled in our study. Plasma and hair samples, total bile acid levels (TBA), alanine transaminase levels (ALT), aspartate aminotransferase levels (AST), and additional clinical information were collected during the third trimester. Metabolites from maternal plasma and hair segments collected pre-conception and analyzed using gas chromatography–mass spectrometry (GC-MS).

**Results:**

Three plasma metabolites (p < 0.05, q < 0.38) and 21 hair metabolites (p < 0.05, q < 0.05) were significantly different between ICP and healthy pregnancies. A combination of the eight most significant hair metabolites in a multivariate receiver operating characteristic curve model showed the best area under the curve (AUC) was 0.885, whereas the highest AUC using metabolites from plasma samples was only 0.74. Metabolic pathway analysis revealed 32 pathways were significantly (p and q values < 0.05) affected in the hair samples of patients with ICP. Pathways associated with glutathione metabolism and ABC transporters were affected. No metabolic pathways were significantly affected in plasma.

**Discussion:**

Overall, this study showed that the hair metabolome could be more useful than the plasma metabolome for distinguishing ICP from normal pregnancy.

## Graphical Abstract

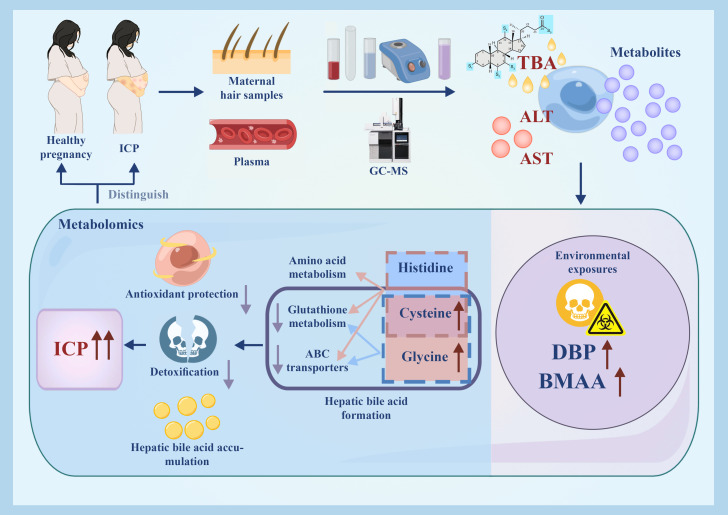

## 1 Introduction

Intrahepatic cholestasis of pregnancy (ICP) is a liver disease which usually manifests in the second or third trimester of pregnancy (1). The diagnosis of ICP is characterized by maternal pruritus as well as elevated concentration of total bile acids and liver transaminases during gestation. In some cases, nausea, vomiting, loss of appetite, or jaundice also occur in ICP patients (2). Although the adverse symptoms of ICP often present low risk to the pregnant women, ICP is categorized as a high-risk perinatal disease because it has been associated with adverse perinatal complications such as fetal hypoxia, premature delivery, growth restriction, fetal distress, meconium-stained amniotic fluid, and stillbirth (3, 4). The incidence of ICP varies globally (0.2%-20%), with particularly high incidence in Chile, Sweden, and China (4, 5). In the Chongqing municipality of China, the incidence of ICP can be as high as 10% (6). Although ICP symptoms and biochemical abnormalities disappear after delivery, subsequent pregnancies are at risk of reoccurrence.

The pathogenesis of ICP is generally perceived to be caused by abnormal biliary tract transportation, which may be related to heredity, sex hormone decompensation, immune dysfunction during pregnancy, environmental influences, or a gene mutation (2–4, 7, 8). There are also reports of a higher incidence in winter, which may be due to lower natural selenium levels or a possible decrease in vitamin D (8–11). Furthermore, there are a number of studies which have investigated bile acid profiles as well as other compounds including progesterone, steroids, and glucose in the blood of ICP patients (12–16). Recently, Cui et al. (2018) studied the serum bile acids of 55 healthy pregnant women, 42 women with ICP and 11 ICP patients treated with ursodeoxycholic acid (UDCA). The ICP group showed a significant difference in the levels of serum bile acids, compared with the control group (13). To our knowledge, untargeted metabolite profiling of blood from women with ICP has not been previously investigated. However, hair was a stable, easy to obtain, and non-invasive biospecimen, has been investigated for specific metabolites of ICP development (17, 18). de Seymour et al. (2018)detected 105 metabolites in hair but none were significantly associated with ICP (18). The differences in metabolite profiles from women with ICP have not yet been compared in both maternal plasma and hair and the exact etiology underlying ICP still remains unclear.

In this study, we investigated the maternal plasma and hair metabolomes from both women diagnosed with ICP and those with a healthy pregnancy, to discover potential specific metabolites and the underlying metabolic changes that occur with ICP development.

## 2 Methods

### 2.1 Study participants and selection criteria

**Figure 1:**
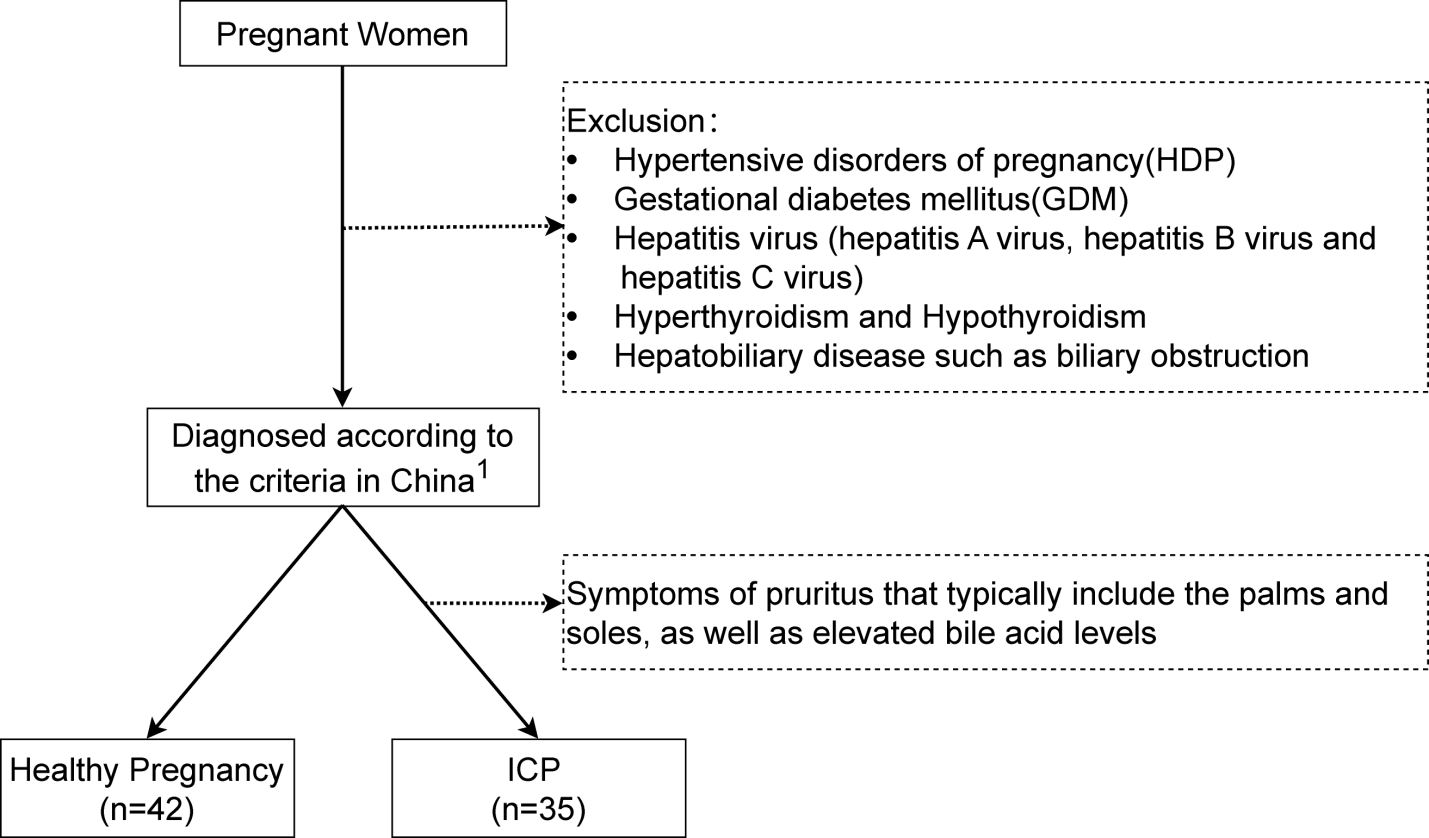
Flowchart of study participants. ^1^The diagnosis of Intrahepatic Cholestasis of Pregnancy (ICP) is characterized by the presence of pruritus with elevated fasting serum total bile acid levels (≥10 μmol/L).

Participants were recruited from the First Affiliated Hospital of Chongqing Medical University, China. A total of 35 cases diagnosed with ICP and 42 controls with a healthy pregnancy were enrolled in this study from January 2018 to October 2019. ICP was diagnosed according to the criteria in China (19). Patients recruited for diagnosing ICP were within the gestational period of 35 + 5 weeks to 41 + 1 weeks. Most cases were mild ICP and presented with late-onset ICP. Laboratory tests, including serum total bile acid (TBA) measurement, served as the primary diagnostic approach for ICP. Moreover, liver function tests, virological examinations, and liver ultrasounds were performed to exclude potential liver and gallbladder conditions or infections. These diagnostic tests were predominantly conducted in the outpatient department, with examination reports reviewed and treatment plans prescribed by, on average, at least one outpatient and one inpatient department physician each. Patients with a hepatitis virus (hepatitis A virus, hepatitis B virus and hepatitis C virus), gestational diabetes mellitus, multiple pregnancies, hyperthyroidism, hypothyroidism, or hepatobiliary disease such as biliary obstruction were excluded from our studies as part of the differential diagnosis for ICP. Additionally, pregnant women with significant skin excoriations caused by trauma, or those receiving oral or topical anti-itch medications during pregnancy, were also excluded from our study (**Figure 1**). The case and control groups were matched for maternal age, pre-pregnancy body mass index (BMI), BMI at delivery, cigarette smoking, alcohol consumption, and parity. Ethical approval for the study was obtained from the Chongqing Medical University Ethics Committee (2019112) and written informed consent was obtained from each patient.

### 2.2 Sample collection

Maternal hair and blood samples were collected from participants several days prior to delivery. Maternal hairs were cut from the occipital area (0.5cm away from the scalp) wrapped inside tinfoil, and the proximal end was labelled. Hair with cosmetic treatment were excluded from our study and all of the pregnant women were ethnic Han Chinese with dark hair. All hair samples were stored at -20°C prior to hair digestion and experienced only a single freeze-thaw cycle prior to analysis. A 7-10 ml blood sample was collected from each participant and transferred into EDTA blood collection tubes (BD Vacutainer) then centrifuged at 3000 rpm for 10 min at 4°C. The supernatants were aliquoted into three Eppendorf tubes (2 mL each). All processed plasma samples were stored at -80°C until sample preparation for analysis.

### 2.3 Hair sample preparation

Both hair and plasma samples were ordered in a randomized sequence prior to sample preparation. Hair samples were cut into three segments (each segment 3cm long). Previous studies have demonstrated that utilizing a 3 cm segmentation of the same hair strand is proficient in discriminating between pregnancy trimesters (20, 21). Thus, the first segment (0-3 cm) represented the third trimester, and the next 0.5 cm hair segment was discarded. Subsequently, the second segment (3.5-6.5 cm) represented the second trimester, the next segment was also discarded, and the third segment (7–10 cm) represented the first trimester. In this study, the first segment was used for research, because intrahepatic cholestasis of pregnancy (ICP) always happened in the third trimester of pregnancy. Hair samples were washed in 2 ml distilled water and 2 ml methanol (Sigma, USA) followed by a 30 s vortex, repeating twice. Hair samples were weighed and between 5-6 mg of hair was segmented and placed into glass vials followed by the addition of 400 µl of 1 M potassium hydroxide (KOH) and 20 µl of 10 mM 2,3,3,3-d4 alanine. Hair samples were incubated at 54°C for 30 min, followed by centrifugation at 4000rpm for 5 min to ensure all the hair samples were completely immersed in the solvent. Samples were then placed in a 54°C incubator for 18 h to complete alkaline hydrolysis of the hair. To neutralize the KOH, a 67ul aliquot of sulphuric acid was added. Methanol (1 ml) was added to remove salts and other residues, followed by centrifugation at 4000rpm for 5 min. A 350 µl of the supernatant from each sample was transferred into a glass tube and the remaining supernatant was pooled into a 50 ml Eppendorf tube to produce a quality control mixture. Four negative controls were prepared using the identical procedure without inclusion of a hair sample. All aliquots were dried in a speedvac concentrator (Savant SPS121P Speedvac, Thermo) at 37°C for 8 h before derivatization. Quality control (QC) samples were also prepared by combining small aliquots of all hair extracts and following the same preparation procedures to the samples.

### 2.4 Plasma sample preparation

400 µL of cold methanol and 20μL of 2,3,3,3-d4 alanine (10 mM) were added to the 100 µL plasma aliquots, followed by vortexing for 1 min and freezing at -20°C for 30 min. The precipitated protein was then eliminated by centrifugation at 12,000 rpm for 15 min at 4°C. A 350 µl aliquot of the supernatant was collected and evaporated to dryness using a speedvac concentrator (Savant SPS121P Speedvac, Thermo) at 37°C for 8h before derivatization.

### 2.5 Methyl chloroformate derivatization

Methyl chloroformate (MCF) derivatization was performed based on the protocol published by Smart et al. (2010) (22). All samples were resuspended in 200μL of 1 M sodium hydroxide and vortexed for 30 s. 167µL of methanol and 34µL of pyridine were added. The derivatization began with the addition of 20µL MCF followed by a 30 s vortex, and then a further 20µL MCF was added, followed by an immediate 30 s vortex. Chloroform (400µL) was added and the samples were mixed for 10 s, followed by the addition of 400µL of 50mM sodium bicarbonate and a 10 s vortex. Samples underwent centrifugation at 2000 rpm for 10 min. The upper aqueous layer was discarded and sodium sulfate added to remove the remaining water before being transferred into gas chromatography (GC) vials for gas chromatography- mass spectrometry (GC-MS) analysis.

### 2.6 GC-MS analysis

The MCF derivatized extracts were analyzed in an Agilent C7890B gas chromatograph coupled to a MSD5975 mass spectrometer. The mass spectrometer setting was at 70 eV. All hair samples were analyzed in a single batch and the gas chromatographic column used for analysis was a zb-1701 gas chromatographic capillary column constituted of 30 m × 250 μm id × 0.15 μm with a 5 m guard column (Phenomenex). GC–MS analysis parameters were based on the parameters reported in Smart et al. (2010) (22) and Han et al. (2019) (23). 1 μL of sample was injected into the inlet using an autosampler with the inlet temperature at 260°C and in the pulsed splitless mode at 180 kPa for 1 min. The helium gas flow through the GC-column was set at a constant flow of 1.0 mL/min. The GC oven temperature was maintained at 45°C for 2 min, and increased at 9°C/min to 180°C held for 5 min. The temperature gradually increased at 40°C/min to 220°C. After 5 minutes, the temperature increased at 40°C/min to 240°C for 11.5 min. Finally, the temperature was increased at 40°C/min to 280°C, and maintained at 280°C for 2 min. The temperature of the interface was kept at 250°C, and the quadrupole temperature was kept at 230°C. The mass spectrum started after 5.5 min with a mass range of 38-550amu, and the scanning time was 0.1 s.

### 2.7 Statistical analysis

Automated Mass Spectral Deconvolution & Identification System (AMDIS) software (version 2.66) was used to deconvolute the GC-MS chromatograms and identify metabolites using our in-house MCF mass spectral library. The compound identifications were based on matching both MS spectrum and the respective chromatographic retention time to compounds in our in-house MCF mass spectral library. The relative abundance of the metabolites was calculated out by Xcalibur (Thermo) by integration and using the GC base-peak value of a specific reference ion. These values were normalized by the abundance of the internal standard (2,3,3,3-d4-alanine), QC samples (three QC samples per batch) and the hair weight. Then, blank samples were utilized to minimize background contamination from identified metabolites. SPSS v22 (SPSS Inc., Chicago, IL) was used for statistical analysis. The student t-tests and Mann Whitney U-test were used to compare differences between the two groups for parametric or non-parametric data respectively. Chi square test was used to compare differences between categorical variables including cigarette smoking, alcohol consumption, and parity. To further identify the metabolic pathways associated with ICP, the metabolites were first annotated into pathways using the KEGG pathway database and their metabolic activities were calculated based on number of identified metabolites in a pathway and their levels, using the Pathway Activity Profling R package (PAPi) (24). The significance for metabolite profile and KEGG analysis was determined by pairwise comparison via Tukey HSD Test.

Receiver operating characteristic (ROC) curves and the area under the ROC curve (AUC) for the relative quantification of hair metabolome were calculated using pROC R package (25). ROC curves established using multiple metabolites were performed with MetaboAnalyst using a linear SVM model (26). Correlations between hair metabolites and TBA concentrations were performed using Pearson’s correlation coefficient. The illustration of heatmaps, line plots, and chord plots were generated using the ggplot2 R packages (27).

## 3 Results

### 3.1 Clinical characteristics of study participants

The clinical characteristics of study participants are shown in **Table 1**; **Supplementary Table 1**. There were no significant differences in age, pre-pregnancy BMI, BMI at delivery, gestational weeks(delivery), cigarette smoking, drinking or parity between case and control groups. The concentrations of TBA, ALT and AST in ICP patients were significantly higher compared to those in the control group.

**Table 1:** Clinical data of the participants.

Maternal Characteristics	Controls Group(n=42)	ICP Group (n=35)	P-value
^b^Maternal age (year)	29 (27, 32)	29 (27, 32)	0.455
^a^Maternal pre-pregnancy BMI (kg/m^2^)	20.77 ± 2.37	20.71 ± 2.20	0.900
^a^Maternal BMI at delivery (kg/m^2^)	26.74 ± 2.44	26.08 ± 2.44	0.241
^a^Gestational age (week)	39.12 ± 0.91	38.41 ± 1.48	0.700
^b^TBA (µmol/l)	1.90 (1.60, 2.30)	17.90 (12.00, 34.30)	< 0.001^***^
^b^ALT (U/l)	17.00 (12.75, 19.75)	43.00 (25.00,152.00)	< 0.001^***^
^b^AST (U/l)	18.50 (15.75, 22.00)	55.90 (23.00, 101.00)	< 0.001^***^

a Clinical data with a normal distribution are expressed as mean ± standard deviation (SD) and a student’s T-test was performed to test for differences between groups;

b Clincal data with non-normal distribution are expressed as median (lower quartile, upper quartile) and a Mann Whitney test was performed to test for differences between groups; ^***^p-value < 0.001.

### 3.2 Metabolomic profiling of hair and plasma

**Figure 2:**
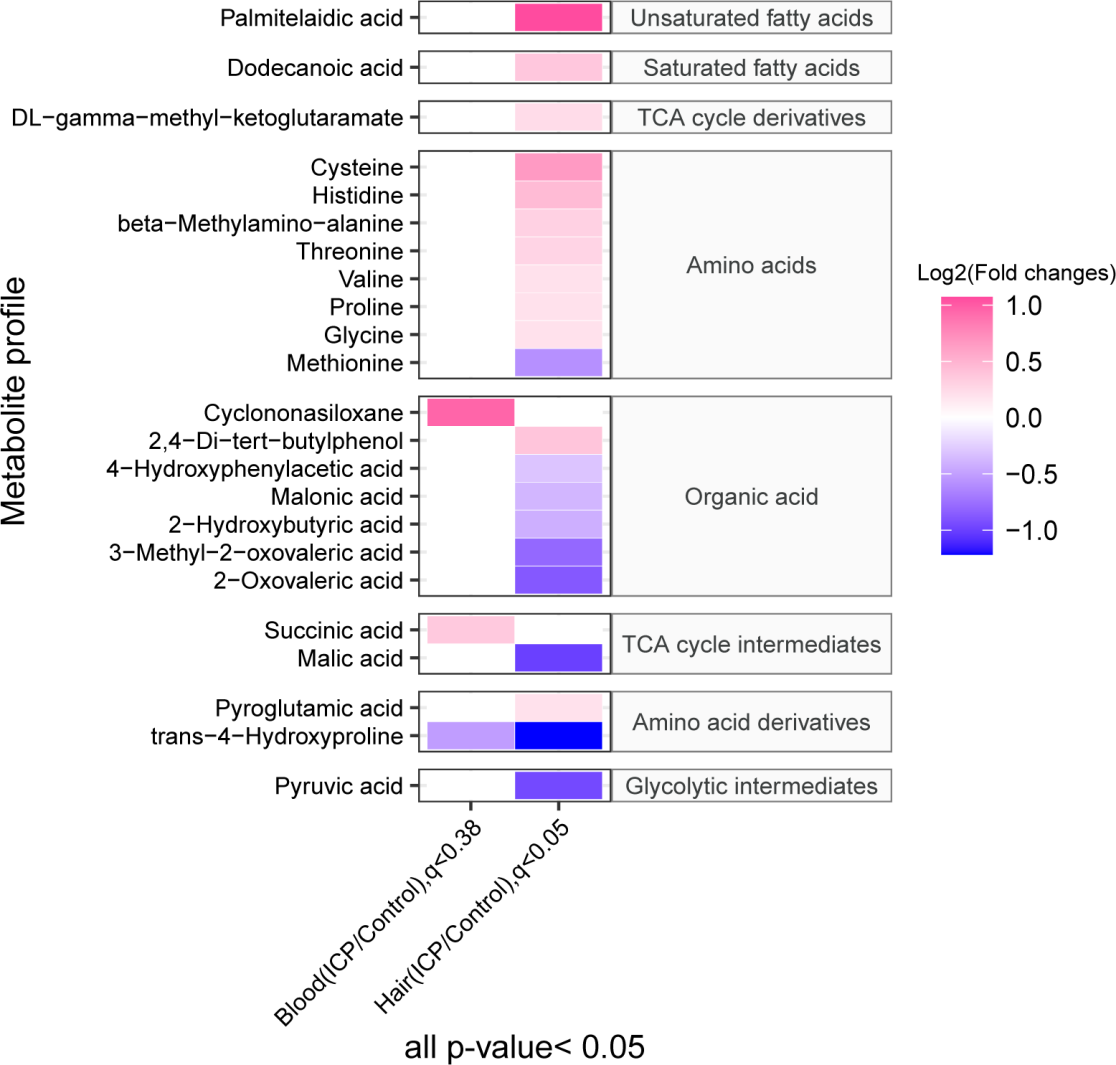
The heatmap demonstrates the maternal plasma and hair metabolome profiles in ICP and control groups. The concentration of metabolites was plotted on a log_2_ scale. Red rectangles represent metabolites with higher levels in the ICP group, while green rectangles represent metabolites with lower levels in the ICP group. Only metabolites with p-values less than 0.05 as well as q-value less than 0.38 or 0.05 for blood and hair samples respectively are shown.

A total of 164 and 155 metabolites were identified in the hair and plasma samples respectively (**Figure 2**; **Supplementary Figure 1**) using our in-house MCF mass spectral library with the inter-assay coefficient of variation in QC samples ranging from 1.1% to 29.6% (See **Supplementary Table 6**). Among them, three plasma metabolites (p-value < 0.05, q-value < 0.38) and 21 hair metabolites (p-value < 0.05, q-value < 0.05) were significantly different between cases and controls. The metabolites identified as significant in the hair included a series of amino acids, unsaturated fatty acids, a saturated fatty acid, organic acids, TCA cycle derivatives, TCA cycle intermediates, amino acid derivatives and glycolytic intermediates. In plasma, the significant metabolites included one organic acid, one TCA cycle intermediate, and one amino acid derivative. Noticeably, the majority of amino acids and fatty acids were in a higher concentration in the ICP group, while most of the organic acids were in a lower concentration in the hair samples of the ICP group (except 2,4-di-tert-butylphenol, BDP).

### 3.3 Receiver operating characteristic curve analysis

**Figure 3:**
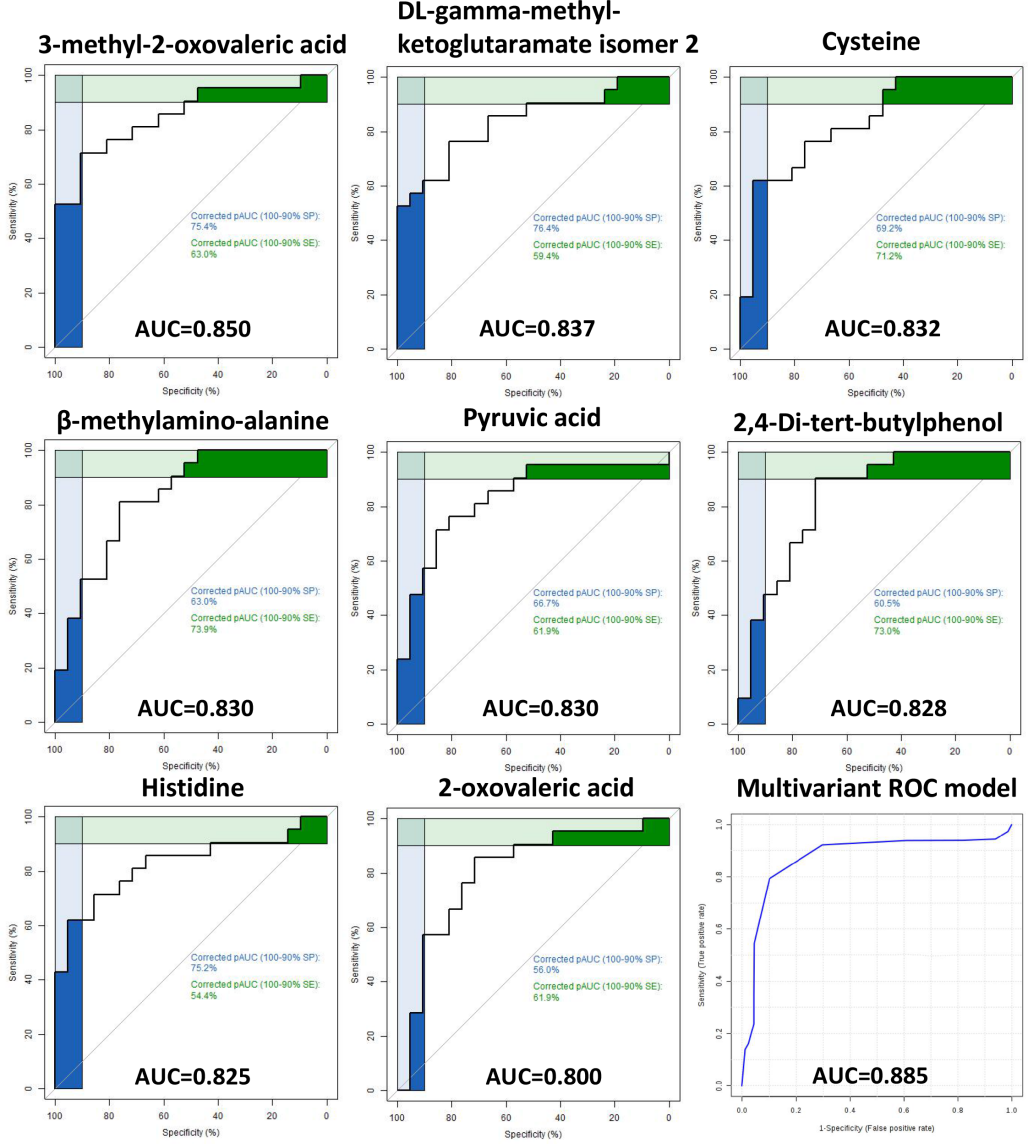
Receiver operating characteristic (ROC) curves of hair metabolites Receiver operating characteristic (ROC) curves of eight metabolites with area under curve (AUC) ≥ 0.80 for predicting ICP using hair samples. A multivariate ROC curve model combining all eight metabolites are shown in the lower right image (AUC of 0.885).

The ROC curve analysis of the metabolites identified in hair and plasma found eight hair metabolites with an AUC above 0.80 for discriminating ICP from healthy pregnancies (**Figure 3**). These comprised of three organic acids (3-methyl-2-oxovaleric acid, beta-methylamino-alanine and 2-oxovaleric acid), two amino acids (cysteine and histidine), one TCA cycle derivative (DL-gamma-methyl-ketoglutaramate isomer 2), and one glycolytic intermediate (pyruvic acid). By combining these eight metabolites into a multivariate ROC curve model an AUC of 0.885 was generated, indicating their potential to segregate ICP from normal pregnancies (**Figure 3**). The highest AUC for any of the metabolites identified in plasma was 0.74 (**Supplementary Tables 2, 3**).

### 3.4 Correlations between metabolites and TBA concentrations

**Figure 4:**
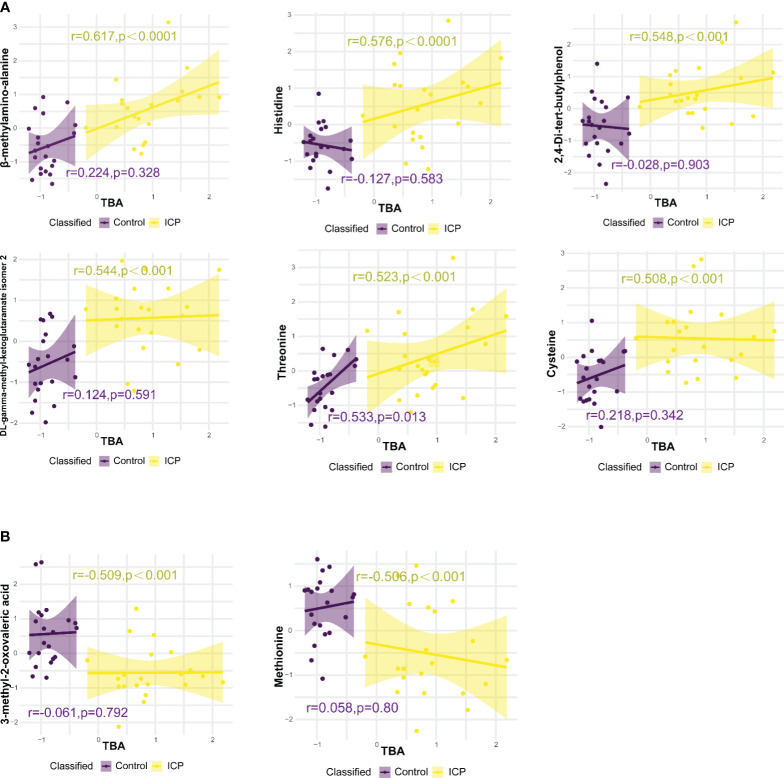
Correlations between hair metabolite levels and total bile acid (TBA) levels, analysed using Pearson correlation. **(A)** Hair metabolites positively correlated with TBA levels. **(B)** Hair metabolites negatively correlated with TBA levels. Only metabolites with p-value < 0.05 and correlation coefficient (r) > 0.5 or < -0.5 are displayed, r and p values for control and ICP groups are represented by purple and yellow colors respectively.

Eight metabolites were significantly correlated with TBA levels based on the following three criteria: 0.5 ≤ r ≤-0.5; p-value < 0.05; and q-value < 0.05. Among them, there were six metabolites positively correlated with TBA levels: beta-methylamino-alanine, histidine, 2,4-Di-tert-butylphenol, threonine, cysteine, and DL-gamma-methyl-ketoglutaramate isomer 2 (**Figure 4A**). Whereas, two hair metabolites were negatively correlated with TBA levels: 3-methyl-2-oxovaleric acid and methionine (**Figure 4B**). No significant correlations were observed between plasma metabolites and TBA levels (**Supplementary Tables 4, 5**).

### 3.5 Predicting metabolic pathway activity

**Figure 5:**
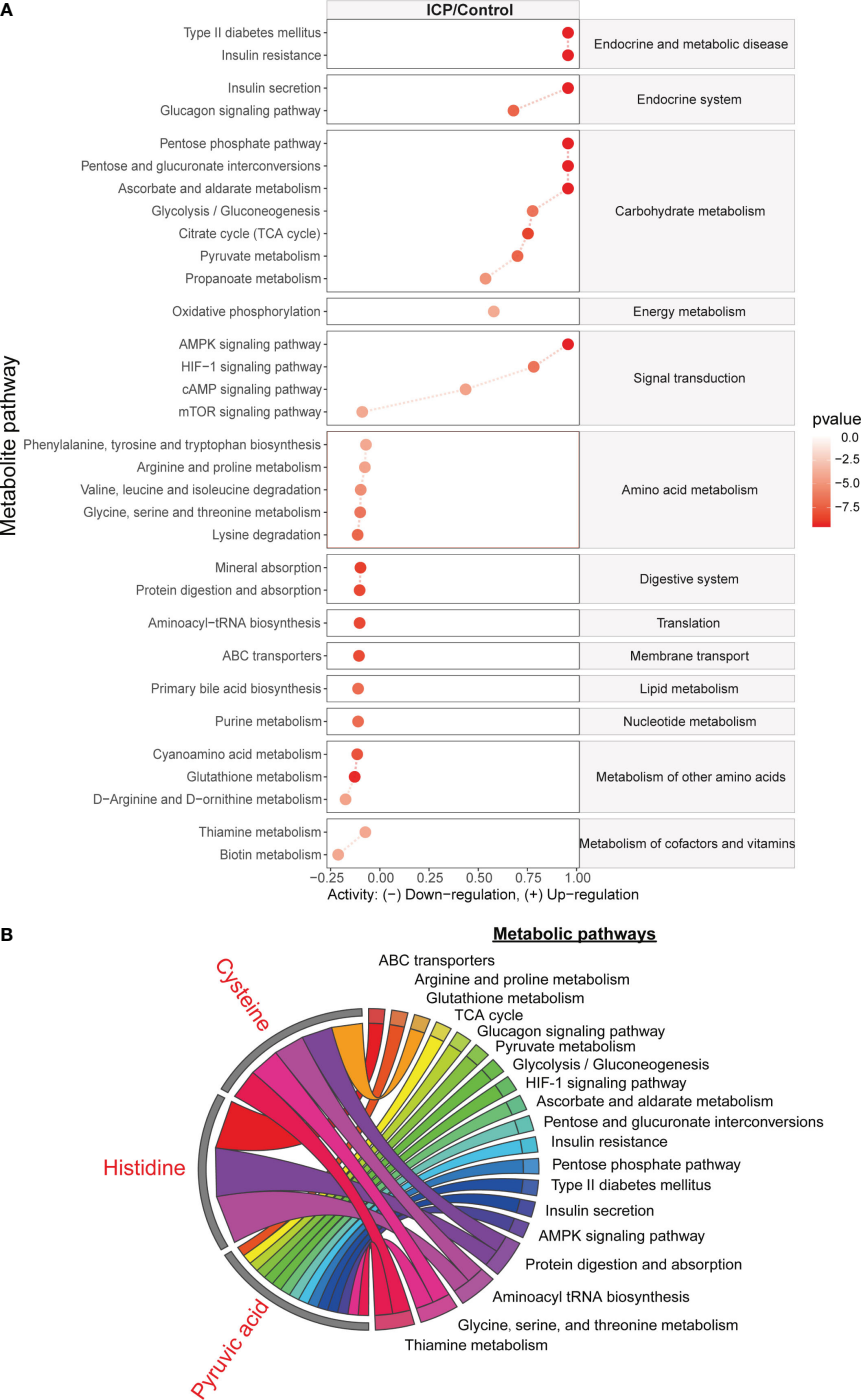
The metabolic pathways significantly associated with ICP, based on metabolites identified in the hair metabolome. **(A)** The line plot indicates the significant metabolic pathways from the analysis using metabolites identified in hair samples. Red lines reflect the metabolic activity in the ICP group. Blue lines reflect the metabolic activity in the control group which have been normalized to zero. The degree of enrichment of the metabolites in the pathway is plotted using a log_2_ scale. Only the metabolic pathways with p-value and q-value less than 0.05 are shown. **(B)** The chord plot displays how three of the significant metabolites (pyruvic acid, cysteine, and histidine) were involved in the different significant metabolic pathways.

A total of 32 metabolic pathways were found to be significantly altered in the hair metabolome of women with ICP pregnancies (p and q values < 0.05) (**Figure 5A**), while no metabolic pathways were significantly different in the plasma samples between cases and controls (all q-values above 0.65). We found the metabolic pathways associated with endocrine and metabolic disease, the endocrine system, carbohydrate metabolism, signal transduction, and energy metabolism were upregulated in ICP (except mTOR signaling pathway). Meanwhile, the metabolic pathways associated with amino acid metabolism, the digestive system, translation, membrane transport, lipid metabolism, nucleotide metabolism, cofactor/vitamin metabolism, and other amino acids metabolism were downregulated in ICP. Furthermore, we have illustrated in **Figure 5B** how three significant metabolites (pyruvic acid, cysteine, and histidine) with AUC of the ROC curve >0.8 and correlation to TBA (r < 0.45, p < 0.05) participated in the previously listed metabolic pathways. The metabolic pathway enrichment analysis indicated that cysteine was involved in five significant pathways, including thiamine metabolism, glycine, serine and threonine metabolism, aminoacyl-tRNA biosynthesis, glutathione metabolism, and protein digestion and absorption; histidine was involved in three significant pathways, namely protein digestion and absorption, aminoacyl-tRNA biosynthesis, and ABC transporters; and pyruvic acid was involved in 15 different metabolic pathways.

## 4 Discussion

In our study, we investigated the maternal plasma and hair metabolomes for differences between women with ICP and those with a healthy pregnancy. Our results indicated that 21 hair metabolites and three plasma metabolites were significantly different between ICP cases and healthy controls. Cysteine and histidine differences observed in the maternal hair between groups were associated with the downregulation of amino acid metabolism, ABC transporters, and glutathione metabolism. Meanwhile, DBP and BMAA in hair were hypothesized to be associated with environmental exposure. Our findings demonstrated that hair could be used to reflect the metabolic perturbations associated with ICP.

The hair metabolites observed in our study to be significantly associated with ICP (in particular, cysteine and glycine) highlighted the importance of glutathione metabolism and ABC transporters on bile formation in ICP. We observed an increased level of cysteine and glycine as well as the downregulation of glutathione metabolism in ICP participants (**Figure 5**). Cysteine and glycine are known to be rate-limiting metabolites for the biosynthesis of glutathione (28). Suppressed catabolism of these sulfur amino acids reduces physiological glutathione level and compromises antioxidant protection and detoxification (29). Wang et al. (2018) also reported that bile acids cause hepatoxicity by reducing the free glutathione pool via suppression of cysteine catabolic pathways in mouse liver (30). Moreover, glutathione contributes to at least one third of bile flow into canaliculi via osmosis, as well as promoting the elimination of harmful substances (e.g. arsenite) along with bile flow (31–33). Not only is glutathione involved in abnormal hepatic bile acid formation, dysregulation of ABC transportation may also contribute to hepatic bile acid accumulation in ICP. Studies have demonstrated that when glutathione-related ABC transportation in mice bile canaliculi was suppressed, the bile flow was also reduced which subsequently promoted the formation of cholestasis (28, 31, 32). Furthermore, we found that ABC transporters were downregulated in ICP patients (**Figure 5A**). Several studies have also reported that the ABC transporters may be related to ICP development. For instance, ABC sub-family B, member 4 (ABCB4) has been found to be related to ICP (2, 4, 34, 35) and dysfunction in ABCB11 has also been identified as a potential risk factor for ICP development (36, 37). Sookoian et al. (2008) reported that an exon 28 of the ABCC2 gene was related to the occurrence of ICP (38). Although our results showed that ABC transporters could transport histidine based on the KEGG database, there are no studies to date that highlight the relationship between ABC transporters and histidine in relation to ICP development. Thus our results in combination with those previously reported supports the conclusion that the accumulation of cysteine, glycine, and histidine in maternal hair may indicate dysregulation of glutathione and ABC transporter metabolism, adversely influencing bile formation in pregnancies affected by ICP.

There were several significant hair metabolites detected in ICP patients that may be associated with environmental pollutants. We observed that 2,4-di-tert-butylphenol (DBP) was significantly elevated in the hair of the ICP cases and that DBP was also positively associated with TBA levels (**Figure 4A**). DBP is a synthetic phenolic antioxidant (SPA) which is widely reported as a toxic compound and environmental contaminant (39). Due to the extensive use of SPAs, these chemicals are prevalent in indoor air, surface water sediment, sludge, dust, and biota as pollutants (40). β-methylamino alanine (BMAA) levels were also found to be associated with ICP, with an AUC of the ROC curve above0.8 and a positive correlation between BMAA with TBA levels (**Figure 4A**). BMAA is a toxin produced by freshwater species and marine cyanobacteria, and may be ingested by humans through water or food (41). No studies to date have reported a relationship between BMAA and ICP. Although previous studies have detected DBP exposure in maternal plasma, cord plasma, placenta, and hair (39, 40), our study is the only one to report a significant association between DBP levels in hair and ICP. Hair can trace exogenous exposure and accumulate environmental contaminants over a period (42), while blood samples change dynamically in response to external stimuli. Thus, the longitudinal profile of hair specimens could reflect exogenous DBP and BMA exposure, which may reflect environmental pollution.

Lastly, our findings showed that pyruvic acid and diabetes-associated metabolic pathways were related to ICP pathogenesis. Although pyruvic acid was involved in fifteen metabolic pathways found to be significantly different between ICP and healthy pregnancies (**Figure 5B**), very few studies have investigated the role of this central metabolite in ICP. Pyruvic acid is not only the end product of the glycolytic pathway, it is also involved in oxidative decarboxylation to form acetyl-coenzyme in the mitochondria, an important part of the TCA cycle (43). *Caldez et al.* (2018) revealed that mitochondrial impairment and TCA cycle downregulation led to hyperactivity of alanine transaminase and a favoring of anaerobic respiration which caused a NAD^+^/NADH redox imbalance in the liver (44). However, the exact role of pyruvic acid in the etiology of ICP requires further investigation.

Despite excluding pregnant women with diagnosed gestational diabetes mellitus (GDM), many of the metabolic pathways we found to be upregulated in ICP were related to type II diabetes mellitus, insulin resistance, and insulin secretion. Studies have shown a higher incidence of GDM in women who develop ICP (45, 46). A prospective study comparing the metabolic outcomes of women with ICP to women with uncomplicated pregnancies found that ICP was associated with impaired glucose tolerance (47). Thus, our results suggest that women who develop ICP during pregnancy may be prone to developing diabetes postpartum. Limitations of this study include a small sample size and lack of multiple time points as well as the hair growth rate varies from person to person, and genetic predispositions, hormonal fluctuations, and external influences may contribute to variations in the pace at which hair grows (48–50). We attempted to minimize this issue by only collecting hair samples from the Han Chinese ethnicity and excluded patients who underwent hair cosmetic treatments. Future research is required to verify our findings, such as the role of glutathione metabolism and ABC transporter-related metabolic pathways in ICP, using a larger cohort and animal models, as well as to investigate the metabolic interrelationships inherent in the physiological dynamics between maternal and offspring entities throughout pregnancy and the postpartum period.

## 5 Conclusion

In summary, we demonstrated that GC-MS based untargeted hair metabolomics was able to discriminate women with ICP from healthy controls, while plasma only displayed minimal differences between groups. The accumulation of hair metabolites, including cysteine and histidine, may indicate the dysregulation of glutathione metabolism and ABC transporters in pregnancies diagnosed with ICP. In addition, the hair metabolome appeared to reflect the accumulation of some environmental exposures (DBP and BMAA), which may be contributing to the pathophysiology of ICP. Overall, the maternal hair metabolome has potential as a non-invasive biospecimen for the diagnosis of ICP and future studies of the exposome related to ICP development.

## Data availability statement

The raw data supporting the conclusions of this article will be made available by the authors, without undue reservation.

## Ethics statement

The studies involving humans were approved by Chongqing Medical University Ethics Committee. The studies were conducted in accordance with the local legislation and institutional requirements. The participants provided their written informed consent to participate in this study.

## Author contributions

NY: Data curation, Formal analysis, Investigation, Methodology, Writing – original draft, Writing – review & editing. XJ: Data curation, Formal analysis, Investigation, Validation, Writing – original draft, Writing – review & editing. MY: Data curation, Formal analysis, Software, Validation, Writing – original draft, Writing – review & editing. YY: Formal analysis, Software, Writing – review & editing. HG: Software, Writing – review & editing. T-LH: Conceptualization, Funding acquisition, Project administration, Supervision, Visualization, Writing – review & editing. HQ: Conceptualization, Funding acquisition, Project administration, Supervision, Visualization, Writing – review & editing.
