# Supplementary material for: The maternal hair metabolome is capable of discriminating intrahepatic cholestasis of pregnancy from uncomplicated pregnancy
Source: Front Endocrinol (Lausanne). 2024 Jan 8;14:1280833. doi: 10.3389/fendo.2023.1280833 (PMC10801165; doi:10.3389/fendo.2023.1280833)
Supplement: Supplementary file 1 [file DataSheet_1.docx]

**Supplementary Table 1.**

Clinical data of the participants

| **Maternal**  **Characteristics** | **Controls**  **Group(n=42)** | **ICP**  **Group (n=35)** | P-value |
| --- | --- | --- | --- |
| ^c^Cigarette smoking  Yes  No | 0 (0.0)  42 (100.0) | 0 (0.0)  35 (100.0) | 1.000 |
| ^c^Alcohol consumption  Yes  No | 0 (0.0)  42 (100.0) | 0 (0.0)  35 (100.0) | 1.000 |
| ^c^Parity  Han nationality  Other nationality | 42 (100.0)  0 (0.0) | 35 (100.0)  0 (0.0) | 1.000 |

^c^Chi square test was used to compare categorical data.

**Supplementary Table 2.** The AUC, p-values, and q-values of all identified metabolites in hair samples.

| name | AUC^a^ | p-value^b^ | q-value^c^ |
| --- | --- | --- | --- |
| 3-Methyl-2-oxopentanoic acid | 0.850 | ＜0.001 | 0.002 |
| DL-gamma-methyl-ketoglutaramate isomer 2 | 0.837 | ＜0.001 | 0.002 |
| Cysteine | 0.832 | ＜0.001 | 0.002 |
| Pyruvic acid | 0.830 | 0.001 | 0.011 |
| beta-Methylamino-alanine | 0.830 | ＜0.001 | 0.002 |
| 2,4-Di-tert-butylphenol | 0.828 | ＜0.001 | 0.002 |
| Histidine | 0.825 | ＜0.001 | 0.002 |
| 2-Oxovaleric acid | 0.800 | 0.001 | 0.011 |
| Methionine | 0.798 | ＜0.001 | 0.005 |
| Malic acid | 0.791 | 0.006 | 0.023 |
| 2-Oxobutyric acid | 0.791 | 0.084 | 0.156 |
| Dodecanoic acid | 0.776 | 0.009 | 0.033 |
| 4-Hydroxyphenylacetic acid | 0.773 | 0.004 | 0.020 |
| Proline | 0.771 | 0.003 | 0.019 |
| Tryptophan | 0.760 | 0.078 | 0.152 |
| trans-4-Hydroxyproline | 0.748 | 0.002 | 0.016 |
| Valine | 0.739 | 0.009 | 0.033 |
| Threonine | 0.739 | 0.004 | 0.020 |
| 2-Hydroxybutyric acid | 0.726 | 0.016 | 0.050 |
| Pentadecanoic acid | 0.719 | 0.019 | 0.055 |
| Palmitelaidic acid | 0.719 | 0.007 | 0.026 |
| Glycine | 0.717 | 0.006 | 0.023 |
| Malonic acid | 0.705 | 0.015 | 0.047 |
| Pyroglutamic acid | 0.701 | 0.013 | 0.045 |
| Putrescine | 0.701 | 0.199 | 0.264 |
| Phenylalanine | 0.701 | 0.027 | 0.068 |
| cis-Aconitic acid | 0.692 | 0.025 | 0.066 |
| Benzoic acid | 0.683 | 0.025 | 0.066 |
| Succinic acid | 0.678 | 0.034 | 0.083 |
| Lysine | 0.678 | 0.047 | 0.104 |
| Ornithine | 0.673 | 0.038 | 0.093 |
| Leucine | 0.673 | 0.049 | 0.104 |
| Tridecanoic acid | 0.667 | 0.160 | 0.229 |
| Myristoleic acid | 0.660 | 0.048 | 0.104 |
| Myristic acid | 0.655 | 0.102 | 0.174 |
| Oleic acid | 0.653 | 0.131 | 0.200 |
| Aspartic acid | 0.651 | 0.102 | 0.174 |
| D_Fumaric acid | 0.647 | 0.666 | 0.466 |
| Suberic acid | 0.642 | 0.130 | 0.200 |
| Cabamic acid | 0.639 | 0.114 | 0.190 |
| Adipic acid | 0.639 | 0.089 | 0.163 |
| Isoleucine | 0.635 | 0.177 | 0.246 |
| N-Acetylglutamic acid | 0.628 | 0.242 | 0.281 |
| Glutaric acid | 0.626 | 0.082 | 0.156 |
| 10-Heptadecenoic acid | 0.626 | 0.096 | 0.172 |
| Citric acid | 0.624 | 0.210 | 0.267 |
| Citraconic acid | 0.624 | 0.328 | 0.306 |
| 10,13-dimethyltetradecanoic acid | 0.621 | 0.126 | 0.200 |
| Pentadecane | 0.619 | 0.988 | 0.555 |
| 9-Heptadecenoic acid | 0.619 | 0.099 | 0.174 |
| Stearic acid | 0.612 | 0.262 | 0.281 |
| Oxaloacetic acid | 0.612 | 0.282 | 0.281 |
| Nicotinic acid | 0.612 | 0.271 | 0.281 |
| D_Norvaline | 0.612 | 0.397 | 0.340 |
| Oxalic acid | 0.610 | 0.279 | 0.281 |
| Norleucine | 0.605 | 0.127 | 0.200 |
| Heneicosanoic acid | 0.605 | 0.308 | 0.300 |
| Asparagine | 0.605 | 0.807 | 0.500 |
| Norvaline | 0.601 | 0.157 | 0.229 |
| Salicylic acid | 0.599 | 0.269 | 0.281 |
| NADP_NADPH | 0.596 | 0.276 | 0.281 |
| Margaric acid | 0.594 | 0.243 | 0.281 |
| beta-Alanine | 0.594 | 0.205 | 0.264 |
| 2-Isopropylmalic acid | 0.594 | 0.223 | 0.277 |
| Itaconic acid | 0.592 | 0.938 | 0.544 |
| Tyrosine | 0.587 | 0.163 | 0.229 |
| 4-Methyl-2-oxopentanoic acid | 0.587 | 0.271 | 0.281 |
| Serine | 0.585 | 0.295 | 0.291 |
| 10-Pentadecenoic acid | 0.585 | 0.265 | 0.281 |
| Dodecane | 0.583 | 0.259 | 0.281 |
| Alanine | 0.583 | 0.188 | 0.258 |
| 16.7644 min N-(Carboxymethyl)-L-alanine | 0.580 | 0.327 | 0.306 |
| Lactic acid | 0.576 | 0.410 | 0.348 |
| Caprylic acid | 0.574 | 0.320 | 0.305 |
| 1-Aminocyclopropane-1-carboxylic acid | 0.574 | 0.414 | 0.348 |
| Glutamic acid | 0.571 | 0.759 | 0.489 |
| 2-Aminobutyric acid | 0.571 | 0.316 | 0.305 |
| Creatinine | 0.569 | 0.353 | 0.311 |
| Citramalic acid | 0.569 | 0.205 | 0.264 |
| 14.9814 min Dimethyl aminomalonic acid | 0.567 | 0.633 | 0.446 |
| Azelaic acid | 0.565 | 0.625 | 0.446 |
| N-alpha-Acetyllysine | 0.562 | 0.556 | 0.414 |
| Dipicolinic acid | 0.562 | 0.490 | 0.391 |
| Tridecane | 0.560 | 0.394 | 0.340 |
| Levulinic acid | 0.558 | 0.715 | 0.475 |
| Hexanoic acid | 0.553 | 0.699 | 0.475 |
| D_Proline | 0.551 | 0.813 | 0.500 |
| 4-Hydroxycinnamic acid | 0.547 | 0.278 | 0.281 |
| gamma-Linolenic acid | 0.546 | 0.561 | 0.414 |
| D_4-Methyl-2-oxopentanoic acid | 0.544 | 0.605 | 0.436 |
| 4-Aminobutyric acid | 0.544 | 0.717 | 0.475 |
| Syringic acid | 0.542 | 0.855 | 0.515 |
| 2-Oxoglutaric acid OR tert-Leucine | 0.540 | 0.481 | 0.390 |
| 2-ketoglutamarate | 0.540 | 0.582 | 0.426 |
| Fumaric acid | 0.537 | 0.893 | 0.524 |
| BHT | 0.537 | 0.700 | 0.475 |
| Glutathione | 0.531 | 0.709 | 0.475 |
| Caprinoic acid | 0.526 | 0.245 | 0.281 |
| 3,5-Diiodo-L-tyrosine | 0.524 | 0.732 | 0.475 |
| Nicotinamide | 0.519 | 0.932 | 0.544 |
| Linoleic acid | 0.519 | 0.769 | 0.492 |
| DL-3-Aminoisobutyric acid | 0.519 | 0.977 | 0.553 |
| D_Indole-3-butyric acid | 0.517 | 0.880 | 0.520 |
| 16.2858 min (-)-O-Acetylmalic anhydride | 0.513 | 0.961 | 0.547 |
| 3-(-2-Thienyl)-D-alanine | 0.508 | 0.352 | 0.311 |
| d3- Alanine | 0.494 | 0.551 | 0.414 |
| Hydroxybenzoic acid | 0.478 | 0.812 | 0.500 |
| Heptadecane | 0.478 | 0.725 | 0.475 |
| DBP | 0.478 | 0.540 | 0.413 |
| 2-Hydroxyglutaramic acid MCF1 | 0.449 | 0.158 | 0.229 |

^a^ROC curve analysis; ^b^Student’s T-test; ^c^False discovery rate; AUC: area under the receiver operating characteristic curve

**Supplementary Table 3.** The AUC, p-values, and q-values of all identified metabolites in plasma samples.

| name | AUC | p value | q value |
| --- | --- | --- | --- |
| Succinic acid | 0.739 | 0.006 | 0.358 |
| trans-4-Hydroxyproline | 0.712 | 0.008 | 0.358 |
| Cycloheptasiloxane, tetradecamethyl | 0.696 | 0.017 | 0.386 |
| Cyclononasiloxane, octadecamethyl | 0.694 | 0.017 | 0.386 |
| Pyruvic acid | 0.678 | 0.042 | 0.596 |
| Methylthioacetic acid | 0.678 | 0.029 | 0.554 |
| Creatinine | 0.676 | 0.082 | 0.696 |
| Lactic acid | 0.673 | 0.148 | 0.770 |
| Glutamic acid | 0.671 | 0.090 | 0.696 |
| 3-Hydroxyoctanoic acid | 0.664 | 0.041 | 0.596 |
| DHA | 0.662 | 0.049 | 0.598 |
| 1-Aminocyclopropane-1-carboxylic acid | 0.653 | 0.041 | 0.596 |
| D_Norvaline | 0.651 | 0.060 | 0.639 |
| Decanoic acid | 0.649 | 0.208 | 0.827 |
| d5-phentlalanine | 0.644 | 0.320 | 0.885 |
| 3-Hydroxydecanoic acid | 0.642 | 0.050 | 0.598 |
| 2-Methyloctadecanoic acid | 0.637 | 0.151 | 0.770 |
| N-alpha-Acetyllysine | 0.635 | 0.075 | 0.696 |
| Isocitric acid | 0.624 | 0.146 | 0.770 |
| Glutathione | 0.624 | 0.272 | 0.827 |
| 2,4-Di-tert-butylphenol | 0.624 | 0.062 | 0.639 |
| Tryptophan | 0.621 | 0.142 | 0.770 |
| beta-Methylamino-alanine | 0.619 | 0.253 | 0.827 |
| Adrenic acid | 0.619 | 0.159 | 0.770 |
| Octanoic acid | 0.617 | 0.351 | 0.923 |
| Glutaric acid | 0.617 | 0.086 | 0.696 |
| Undecane, 2-methyl | 0.615 | 0.155 | 0.770 |
| Oxalic acid | 0.615 | 0.381 | 0.949 |
| Azelaic acid | 0.612 | 0.268 | 0.827 |
| Tricosane | 0.610 | 0.206 | 0.827 |
| Nicotinamide | 0.610 | 0.154 | 0.770 |
| Glycine | 0.610 | 0.329 | 0.894 |
| Hippuric acid | 0.608 | 0.486 | 0.977 |
| Dodecanoic acid | 0.608 | 0.391 | 0.949 |
| 9-Heptadecenoic acid | 0.608 | 0.217 | 0.827 |
| DL-3-Aminoisobutyric acid | 0.605 | 0.434 | 0.977 |
| Pyroglutamic acid | 0.601 | 0.210 | 0.827 |
| Glutamine | 0.601 | 0.254 | 0.827 |
| 4-Aminobutyric acid | 0.592 | 0.256 | 0.827 |
| Oleic acid | 0.590 | 0.141 | 0.770 |
| Methionine | 0.590 | 0.242 | 0.827 |
| cis-Vaccenic acid | 0.590 | 0.147 | 0.770 |
| EDTA | 0.587 | 0.537 | 0.977 |
| Dodecane | 0.587 | 0.529 | 0.977 |
| Cysteine | 0.585 | 0.551 | 0.982 |
| 4-Methyl-2-oxopentanoic acid | 0.583 | 0.824 | 0.998 |
| Nicotinic acid | 0.581 | 0.116 | 0.770 |
| 11,14-Eicosadienoic | 0.580 | 0.459 | 0.977 |
| Palmitelaidic acid | 0.574 | 0.542 | 0.977 |
| Norleucine | 0.574 | 0.235 | 0.827 |
| alpha-Linolenic acid | 0.574 | 0.401 | 0.949 |
| Tridecane | 0.571 | 0.518 | 0.977 |
| Leucine | 0.571 | 0.503 | 0.977 |
| gamma-Linolenic acid | 0.565 | 0.307 | 0.881 |
| 2-Hydroxybutyric acid | 0.562 | 0.655 | 0.994 |
| 16.7644 min N-(Carboxymethyl)-L-alanine | 0.560 | 0.579 | 0.994 |
| S-Adenosylmethionine | 0.558 | 0.400 | 0.949 |
| Phenethyl acetate | 0.556 | 0.718 | 0.994 |
| Myristoleic acid | 0.551 | 0.301 | 0.881 |
| Palmitic acid | 0.549 | 0.219 | 0.827 |
| 2-Aminoadipic acid | 0.549 | 0.489 | 0.977 |
| D_Fumaric acid | 0.546 | 0.827 | 0.998 |
| Citraconic acid | 0.546 | 0.761 | 0.998 |
| 4-Hydroxyphenylacetic acid | 0.546 | 0.349 | 0.923 |
| Pentadecane | 0.544 | 0.624 | 0.994 |
| DPA | 0.542 | 0.494 | 0.977 |
| Levulinic acid | 0.540 | 0.486 | 0.977 |
| Arachidonic acid | 0.540 | 0.479 | 0.977 |
| Valine | 0.537 | 0.906 | 0.998 |
| cis-4-Hydroxyproline | 0.535 | 0.897 | 0.998 |
| bishomo-gamma-Linolenic acid | 0.535 | 0.682 | 0.994 |
| BHT | 0.535 | 0.494 | 0.977 |
| Glyoxylic acid | 0.533 | 0.823 | 0.998 |
| Asparagine | 0.533 | 0.599 | 0.994 |
| Norvaline | 0.528 | 0.705 | 0.994 |
| DBP | 0.528 | 0.896 | 0.998 |
| Myristic acid | 0.526 | 0.785 | 0.998 |
| 3-Methyl-2-oxopentanoic acid | 0.526 | 0.977 | 0.999 |
| DL-gamma-methyl-ketoglutaramate isomer 1 | 0.524 | 0.686 | 0.994 |
| beta-Alanine | 0.524 | 0.712 | 0.994 |
| Phenylalanine | 0.522 | 0.911 | 0.998 |
| Propyl gallate | 0.519 | 0.571 | 0.994 |
| Nonadecanoic acid | 0.519 | 0.978 | 0.999 |
| Gondoic acid | 0.519 | 0.911 | 0.998 |
| EPA | 0.519 | 0.955 | 0.999 |
| Alanine | 0.519 | 0.939 | 0.998 |
| Pentadecanoic acid | 0.517 | 0.940 | 0.998 |
| Margaric acid | 0.515 | 0.741 | 0.998 |
| Proline | 0.512 | 0.668 | 0.994 |
| Cabamic acid | 0.512 | 0.878 | 0.998 |
| Benzoic acid | 0.512 | 0.636 | 0.994 |
| 2-Oxoglutaric acid | 0.512 | 0.787 | 0.998 |
| 2-Hydroxyglutaramic acid MCF1 | 0.512 | 0.891 | 0.998 |
| Malic acid | 0.510 | 0.795 | 0.998 |
| Citric acid | 0.510 | 0.931 | 0.998 |
| Isoleucine | 0.508 | 0.879 | 0.998 |
| cis-Aconitic acid | 0.508 | 0.919 | 0.998 |
| Dehydroascorbic acid | 0.506 | 0.992 | 0.999 |
| Erucic acid | 0.499 | 0.996 | 0.999 |
| Arachidic acid | 0.497 | 0.651 | 0.994 |
| Stearic acid | 0.492 | 0.951 | 0.999 |
| Serine | 0.492 | 0.995 | 0.999 |
| 2-Aminobutyric acid | 0.490 | 0.999 | 0.999 |
| Threonine | 0.481 | 0.664 | 0.994 |
| Ornithine | 0.478 | 0.713 | 0.994 |
| Lysine | 0.476 | 0.537 | 0.977 |
| Fumaric acid | 0.476 | 0.795 | 0.998 |
| Malonic acid | 0.474 | 0.272 | 0.827 |
| Tyrosine | 0.465 | 0.486 | 0.977 |
| D_Alanine | 0.463 | 0.755 | 0.998 |
| Histidine | 0.458 | 0.693 | 0.994 |
| Aspartic acid | 0.451 | 0.665 | 0.994 |
| Hexanoic acid | 0.449 | 0.507 | 0.977 |
| Adipic acid | 0.449 | 0.626 | 0.994 |
| Glyceric acid | 0.447 | 0.675 | 0.994 |
| Linoleic acid | 0.442 | 0.215 | 0.827 |
| Itaconic acid | 0.422 | 0.435 | 0.977 |

^a^ROC curve analysis; ^b^Student’s T-test; ^C^False discovery rate; AUC: area under the receiver operating characteristic curve

**Supplementary Table 4.** Correlations between hair metabolites and TBA concentrations

| name | r^a^ | p-value^b^ | q-value^c^ |
| --- | --- | --- | --- |
| beta-Methylamino-alanine | 0.617 | ＜0.001 | ＜0.001 |
| Histidine | 0.576 | ＜0.001 | 0.001 |
| 2,4-Di-tert-butylphenol | 0.548 | ＜0.001 | 0.001 |
| DL-gamma-methyl-ketoglutaramate isomer 2 | 0.544 | ＜0.001 | 0.001 |
| Threonine | 0.523 | ＜0.001 | 0.002 |
| 3-Methyl-2-oxopentanoic acid | -0.509 | 0.001 | 0.002 |
| Cysteine | 0.508 | 0.001 | 0.002 |
| Methionine | -0.506 | 0.001 | 0.002 |
| Proline | 0.494 | 0.001 | 0.002 |
| Valine | 0.476 | 0.001 | 0.004 |
| Glycine | 0.470 | 0.002 | 0.004 |
| Pyruvic acid | -0.464 | 0.002 | 0.004 |
| Dodecanoic acid | 0.448 | 0.003 | 0.005 |
| 4-Hydroxyphenylacetic acid | -0.446 | 0.003 | 0.005 |
| Ornithine | 0.443 | 0.003 | 0.005 |
| trans-4-Hydroxyproline | -0.437 | 0.004 | 0.006 |
| Malic acid 1 | -0.437 | 0.004 | 0.006 |
| 2-Oxovaleric acid | -0.419 | 0.006 | 0.008 |
| Lysine | 0.396 | 0.010 | 0.012 |
| Leucine | 0.383 | 0.012 | 0.015 |
| Pyroglutamic acid | 0.370 | 0.016 | 0.019 |
| 2-Hydroxybutyric acid | -0.360 | 0.019 | 0.022 |
| Malonic acid | -0.351 | 0.023 | 0.024 |
| cis-Aconitic acid | -0.340 | 0.028 | 0.027 |
| Phenylalanine | 0.339 | 0.028 | 0.027 |
| Myristoleic acid | 0.337 | 0.029 | 0.027 |
| Isoleucine | 0.316 | 0.042 | 0.038 |
| Palmitelaidic acid | 0.307 | 0.048 | 0.042 |
| Benzoic acid | -0.303 | 0.051 | 0.043 |
| Adipic acid | 0.302 | 0.052 | 0.043 |
| Pentadecanoic acid | 0.299 | 0.055 | 0.043 |
| beta-Alanine | 0.287 | 0.065 | 0.049 |
| Suberic acid | 0.287 | 0.065 | 0.049 |
| Tryptophan | -0.284 | 0.068 | 0.049 |
| N-Acetylglutamic acid | 0.278 | 0.074 | 0.052 |
| Succinic acid | -0.276 | 0.077 | 0.053 |
| Aspartic acid | 0.274 | 0.079 | 0.053 |
| 10-Heptadecenoic acid | 0.268 | 0.086 | 0.056 |
| Norvaline | 0.256 | 0.102 | 0.063 |
| 9-Heptadecenoic acid | 0.255 | 0.103 | 0.063 |
| D_Fumaric acid | -0.244 | 0.151 | 0.079 |
| 3-(-2-Thienyl)-D-alanine | 0.244 | 0.120 | 0.070 |
| Norleucine | 0.244 | 0.120 | 0.070 |
| 2-Oxobutyric acid | -0.243 | 0.121 | 0.070 |
| Alanine | 0.238 | 0.129 | 0.072 |
| Citraconic acid | -0.234 | 0.135 | 0.074 |
| 1-Aminocyclopropane-1-carboxylic acid | 0.226 | 0.149 | 0.079 |
| Putrescine | 0.217 | 0.167 | 0.083 |
| d3- Alanine | 0.217 | 0.167 | 0.083 |
| 2-Isopropylmalic acid | 0.216 | 0.169 | 0.083 |
| Tyrosine | 0.209 | 0.183 | 0.086 |
| Dodecane | -0.209 | 0.183 | 0.086 |
| Oleic acid | 0.208 | 0.186 | 0.086 |
| Salicylic acid | -0.205 | 0.193 | 0.087 |
| Glutaric acid | 0.203 | 0.197 | 0.087 |
| Tridecanoic acid | 0.202 | 0.199 | 0.087 |
| Serine | 0.197 | 0.211 | 0.091 |
| Heneicosanoic acid | 0.196 | 0.214 | 0.091 |
| Myristic acid | 0.192 | 0.223 | 0.093 |
| Margaric acid | 0.188 | 0.233 | 0.096 |
| 10-Pentadecenoic acid | 0.180 | 0.253 | 0.102 |
| Caprylic acid | -0.167 | 0.292 | 0.115 |
| 16.7644 min N-(Carboxymethyl)-L-alanine | 0.166 | 0.293 | 0.115 |
| Citric acid | -0.161 | 0.309 | 0.118 |
| 2-Aminobutyric acid | 0.159 | 0.316 | 0.118 |
| Lactic acid | -0.158 | 0.316 | 0.118 |
| DL-3-Aminoisobutyric acid | 0.155 | 0.327 | 0.119 |
| 10,13-dimethyltetradecanoic acid | 0.153 | 0.333 | 0.119 |
| Tridecane | -0.153 | 0.333 | 0.119 |
| Citramalic acid | 0.149 | 0.347 | 0.122 |
| 2-Hydroxyglutaramic acid MCF1 | 0.133 | 0.401 | 0.138 |
| 4-Methyl-2-oxopentanoic acid | -0.132 | 0.405 | 0.138 |
| Cabamic acid | -0.130 | 0.413 | 0.138 |
| NADP_NADPH | -0.129 | 0.415 | 0.138 |
| 4-Hydroxycinnamic acid | -0.122 | 0.477 | 0.147 |
| Caprinoic acid | 0.121 | 0.446 | 0.146 |
| DBP | 0.120 | 0.450 | 0.146 |
| BHT | -0.118 | 0.459 | 0.146 |
| Hexanoic acid | -0.116 | 0.465 | 0.146 |
| D_4-Methyl-2-oxopentanoic acid | -0.115 | 0.467 | 0.146 |
| Glutamic acid | 0.110 | 0.489 | 0.147 |
| D_Norvaline | 0.109 | 0.491 | 0.147 |
| Dipicolinic acid | 0.106 | 0.505 | 0.150 |
| 16.2858 min (-)-O-Acetylmalic anhydride | -0.103 | 0.534 | 0.153 |
| gamma-Linolenic acid | -0.100 | 0.529 | 0.153 |
| 4-Aminobutyric acid | 0.100 | 0.530 | 0.153 |
| Oxalic acid | -0.093 | 0.560 | 0.158 |
| Syringic acid | 0.091 | 0.568 | 0.159 |
| Creatinine | -0.089 | 0.573 | 0.159 |
| Azelaic acid | 0.086 | 0.586 | 0.159 |
| Itaconic acid | -0.086 | 0.589 | 0.159 |
| Asparagine | -0.077 | 0.626 | 0.168 |
| Heptadecane | -0.075 | 0.638 | 0.168 |
| Nicotinic acid | -0.074 | 0.642 | 0.168 |
| Linoleic acid | -0.073 | 0.647 | 0.168 |
| 3,5-Diiodo-L-tyrosine | -0.067 | 0.671 | 0.172 |
| Nicotinamide | 0.062 | 0.695 | 0.176 |
| Levulinic acid | -0.061 | 0.701 | 0.176 |
| Oxaloacetic acid | -0.059 | 0.708 | 0.176 |
| N-alpha-Acetyllysine | 0.055 | 0.728 | 0.178 |
| Fumaric acid | -0.055 | 0.731 | 0.178 |
| D_Proline | 0.051 | 0.749 | 0.179 |
| 2-ketoglutamarate | -0.051 | 0.751 | 0.179 |
| Stearic acid | 0.048 | 0.763 | 0.181 |
| Hydroxybenzoic acid | -0.046 | 0.772 | 0.181 |
| Pentadecane | -0.027 | 0.865 | 0.201 |
| 2-Oxoglutaric acid OR tert-Leucine | -0.026 | 0.872 | 0.201 |
| 14.9814 min Dimethyl aminomalonic acid | -0.019 | 0.906 | 0.204 |
| Glutathione | 0.018 | 0.911 | 0.204 |
| D_Indole-3-butyric acid | 0.009 | 0.953 | 0.211 |

^a^Correlation coefficient and ^b^p-value from Pearson correlation; ^c^False discovery rate; TBA:

**Supplementary Table 5.** Correlations between plasma metabolites and TBA concentrations

| name | r | p-value | q-value |
| --- | --- | --- | --- |
| Cycloheptasiloxane, tetradecamethyl | 0.445 | 0.003 | 0.199 |
| Cyclononasiloxane, octadecamethyl | 0.442 | 0.003 | 0.199 |
| Succinic acid | 0.396 | 0.009 | 0.326 |
| D_Norvaline | -0.388 | 0.011 | 0.326 |
| N-alpha-Acetyllysine | 0.359 | 0.020 | 0.369 |
| 2,4-Di-tert-butylphenol | 0.359 | 0.020 | 0.369 |
| Glutaric acid | 0.353 | 0.022 | 0.369 |
| Methylthioacetic acid | 0.326 | 0.035 | 0.464 |
| Pyruvic acid | -0.324 | 0.037 | 0.464 |
| DHA | -0.317 | 0.041 | 0.464 |
| trans-4-Hydroxyproline | -0.313 | 0.043 | 0.464 |
| Nicotinamide | 0.306 | 0.049 | 0.479 |
| Lactic acid | -0.290 | 0.062 | 0.528 |
| Undecane, 2-methyl | 0.290 | 0.063 | 0.528 |
| Malonic acid | 0.267 | 0.087 | 0.687 |
| 3-Hydroxyoctanoic acid | 0.260 | 0.097 | 0.712 |
| Methionine | 0.254 | 0.104 | 0.723 |
| Nicotinic acid | 0.250 | 0.120 | 0.788 |
| 3-Hydroxydecanoic acid | 0.236 | 0.133 | 0.826 |
| Tricosane | -0.225 | 0.152 | 0.866 |
| 4-Hydroxyphenylacetic acid | 0.216 | 0.169 | 0.866 |
| Glutamine | 0.211 | 0.180 | 0.866 |
| 4-Aminobutyric acid | 0.209 | 0.184 | 0.866 |
| Azelaic acid | 0.207 | 0.189 | 0.866 |
| BHT | 0.206 | 0.191 | 0.866 |
| 1-Aminocyclopropane-1-carboxylic acid | -0.202 | 0.199 | 0.866 |
| Norleucine | 0.196 | 0.213 | 0.866 |
| Oxalic acid | 0.195 | 0.216 | 0.866 |
| 2-Methyloctadecanoic acid | 0.193 | 0.220 | 0.866 |
| Glutamic acid | -0.189 | 0.231 | 0.866 |
| Pyroglutamic acid | 0.186 | 0.238 | 0.866 |
| beta-Methylamino-alanine | 0.186 | 0.239 | 0.866 |
| Creatinine | -0.185 | 0.242 | 0.866 |
| 2-Hydroxybutyric acid | -0.180 | 0.255 | 0.870 |
| Decanoic acid | 0.170 | 0.282 | 0.870 |
| Tryptophan | -0.168 | 0.288 | 0.870 |
| Adrenic acid | -0.165 | 0.297 | 0.870 |
| d5-phentlalanine | -0.165 | 0.297 | 0.870 |
| Myristoleic acid | 0.165 | 0.298 | 0.870 |
| 3-Methyl-2-oxopentanoic acid | -0.162 | 0.306 | 0.870 |
| S-Adenosylmethionine | -0.157 | 0.322 | 0.870 |
| 11,14-Eicosadienoic | -0.155 | 0.327 | 0.870 |
| Palmitic acid | 0.146 | 0.355 | 0.870 |
| Oleic acid | 0.146 | 0.357 | 0.870 |
| DPA | -0.144 | 0.361 | 0.870 |
| DL-3-Aminoisobutyric acid | 0.141 | 0.373 | 0.870 |
| cis-Vaccenic acid | 0.141 | 0.373 | 0.870 |
| Isocitric acid | 0.141 | 0.373 | 0.870 |
| Malic acid | 0.140 | 0.377 | 0.870 |
| Histidine | 0.136 | 0.390 | 0.870 |
| Tridecane | -0.134 | 0.396 | 0.870 |
| Threonine | 0.134 | 0.396 | 0.870 |
| Aspartic acid | 0.133 | 0.401 | 0.870 |
| 9-Heptadecenoic acid | 0.132 | 0.406 | 0.870 |
| Benzoic acid | 0.130 | 0.411 | 0.870 |
| Octanoic acid | 0.130 | 0.413 | 0.870 |
| Adipic acid | 0.125 | 0.431 | 0.892 |
| Cysteine | 0.120 | 0.449 | 0.913 |
| Tyrosine | 0.115 | 0.470 | 0.939 |
| Glycine | 0.107 | 0.500 | 0.968 |
| Linoleic acid | 0.106 | 0.505 | 0.968 |
| Itaconic acid | 0.104 | 0.514 | 0.968 |
| alpha-Linolenic acid | -0.099 | 0.533 | 0.968 |
| Glutathione | -0.098 | 0.536 | 0.968 |
| Arachidonic acid | -0.098 | 0.539 | 0.968 |
| Nonadecanoic acid | 0.097 | 0.543 | 0.968 |
| Palmitelaidic acid | 0.095 | 0.549 | 0.968 |
| Levulinic acid | -0.088 | 0.579 | 0.991 |
| Fumaric acid | 0.087 | 0.585 | 0.991 |
| Dodecanoic acid | 0.083 | 0.601 | 0.991 |
| 3-methyl-2-oxovaleric acid | -0.079 | 0.620 | 0.991 |
| 2-Hydroxyglutaramic acid MCF1 | 0.078 | 0.624 | 0.991 |
| Leucine | 0.072 | 0.649 | 0.991 |
| 16.7644 min N-(Carboxymethyl)-L-alanine | 0.072 | 0.651 | 0.991 |
| 2-Aminobutyric acid | -0.070 | 0.658 | 0.991 |
| Hexanoic acid | 0.070 | 0.659 | 0.991 |
| D_Fumaric acid | 0.064 | 0.688 | 0.991 |
| D_Alanine | 0.064 | 0.689 | 0.991 |
| Arachidic acid | -0.063 | 0.692 | 0.991 |
| Erucic acid | -0.056 | 0.723 | 0.991 |
| Asparagine | -0.052 | 0.742 | 0.991 |
| Pentadecane | -0.050 | 0.754 | 0.991 |
| gamma-Linolenic acid | 0.050 | 0.755 | 0.991 |
| Gondoic acid | 0.048 | 0.762 | 0.991 |
| Pentadecanoic acid | -0.046 | 0.771 | 0.991 |
| Ornithine | -0.043 | 0.785 | 0.991 |
| DBP | 0.042 | 0.790 | 0.991 |
| Glyceric acid | 0.041 | 0.797 | 0.991 |
| EPA | 0.040 | 0.799 | 0.991 |
| Citric acid 1 | 0.040 | 0.800 | 0.991 |
| beta-Alanine | 0.040 | 0.803 | 0.991 |
| Isoleucine | -0.039 | 0.807 | 0.991 |
| Dehydroascorbic acid | 0.039 | 0.809 | 0.991 |
| Valine | -0.037 | 0.815 | 0.991 |
| Cabamic acid | -0.036 | 0.821 | 0.991 |
| cis-4-Hydroxyproline | -0.033 | 0.833 | 0.991 |
| Lysine | -0.030 | 0.850 | 0.991 |
| 2-Oxoglutaric acid | -0.029 | 0.856 | 0.991 |
| bishomo-gamma-Linolenic acid | -0.025 | 0.873 | 0.991 |
| Glyoxylic acid | 0.024 | 0.878 | 0.991 |
| Citraconic acid | -0.021 | 0.893 | 0.991 |
| Proline | 0.021 | 0.894 | 0.991 |
| Propyl gallate | -0.021 | 0.895 | 0.991 |
| EDTA | 0.021 | 0.897 | 0.991 |
| Alanine | -0.020 | 0.900 | 0.991 |
| Serine | 0.020 | 0.900 | 0.991 |
| Phenethyl acetate | -0.018 | 0.910 | 0.991 |
| Phenylalanine | -0.013 | 0.933 | 0.991 |
| Myristic acid | -0.010 | 0.952 | 0.991 |
| Margaric acid | -0.009 | 0.954 | 0.991 |
| Norvaline | 0.009 | 0.955 | 0.991 |
| Hippuric acid | -0.008 | 0.959 | 0.991 |
| Stearic acid | 0.008 | 0.959 | 0.991 |
| Dodecane | -0.007 | 0.963 | 0.991 |
| 2-Aminoadipic acid | -0.006 | 0.971 | 0.991 |
| DL-gamma-methyl-ketoglutaramate isomer 1 | 0.005 | 0.977 | 0.991 |
| cis-Aconitic acid | 0.003 | 0.986 | 0.991 |

^a^Correlation coefficient and ^b^p-value from Pearson correlation; ^c^False discovery rate.

**Supplementary Table 6.** Identified metabolites

| **Metabolite names CV (%)** | | |
| --- | --- | --- |
| Dodecane | 3.3 |  |
| Alanine | 3.6 |  |
| d4-Alanine | 3.8 |  |
| Glycine | 3.9 |  |
| D_Proline | 4.2 |  |
| d3- Alanine | 4.3 |  |
| 3-(-2-Thienyl)-D-alanine | 4.8 |  |
| d5-phentlalanine | 4.9 |  |
| 2-Aminobutyric acid | 6.2 |  |
| Phenylalanine | 6.8 |  |
| Leucine | 7.1 |  |
| Isoleucine | 7.3 |  |
| Valine | 7.3 |  |
| Proline | 7.4 |  |
| Tridecane | 7.8 |  |
| Nicotinamide | 7.8 |  |
| DL-gamma-methyl-ketoglutaramate isomer 2 | 8.2 |  |
| Caprinoic acid (split peak 1) OR Decanoic acid (C10_0) | 8.7 |  |
| beta-Alanine | 9.0 |  |
| 16.7644 min N-(Carboxymethyl)-L-alanine | 9.2 |  |
| Dipicolinic acid | 9.2 |  |
| Pyroglutamic acid | 9.3 |  |
| Heptadecane | 9.7 |  |
| Nicotinic acid | 10.5 |  |
| Hydroxybenzoic acid | 11.1 |  |
| 2-Oxoglutaric acid OR tert-Leucine | 11.7 |  |
| 4-Hydroxyphenylacetic acid | 11.7 |  |
| 2-Oxobutyric acid | 12.1 |  |
| 2-Hydroxyglutaramic acid MCF1 OR D_5-Oxotetrahydrofuran-2-carboxylic acid | 12.3 |  |
| Norvaline (split peak 3) | 12.3 |  |
| Pentadecane (split peak 2) | 12.4 |  |
| 2,4-Di-tert-butylphenol (derivatization artefact) | 12.4 |  |
| Tridecanoic acid (C13_0) | 12.8 |  |
| Aspartic acid | 12.8 |  |
| 4-Aminobutyric acid (GABA) | 13.4 |  |
| Citramalic acid | 13.5 |  |
| 3-Methyl-2-oxopentanoic acid OR 3-Methyl-2-oxovaleric acid | 13.9 |  |
| Threonine | 14.0 |  |
| NADP_NADPH | 14.3 |  |
| Citraconic acid | 15.9 |  |
| 10-Pentadecenoic acid (C15_1n-5c) | 16.3 |  |
| BHT (Antioxidant) | 16.6 |  |
| Benzoic acid | 16.6 |  |
| 2-Isopropylmalic acid | 16.9 |  |
| D_Indole-3-butyric acid | 17.1 |  |
| Asparagine | 17.5 |  |
| Levulinic acid | 17.6 |  |
| 1-Aminocyclopropane-1-carboxylic acid | 18.0 |  |
| Azelaic acid | 18.3 |  |
| d2-tyrosine | 18.7 |  |
| D_4-Methyl-2-oxopentanoic acid | 18.7 |  |
| 4-Methyl-2-oxopentanoic acid | 18.7 |  |
| Adipic acid | 18.8 |  |
| cis-Aconitic acid | 19.1 |  |
| Salicylic acid | 19.2 |  |
| gamma-Linolenic acid (C18_3n-6,9,12c) OR alpha-Linolenic acid (C18_3n-3,6,9c) OR Gondoic acid (C20_1n-9c) | 19.5 |  |
| 2-ketoglutamarate | 19.9 |  |
| Stearic acid (C18_0) | 19.9 |  |
| 4-Hydroxycinnamic acid | 20.0 |  |
| Fumaric acid | 20.1 |  |
| Methionine | 20.5 |  |
| Histidine | 20.5 |  |
| Putrescine | 21.0 |  |
| Dodecanoic acid (C12_0) | 21.1 |  |
| 9-Heptadecenoic acid (C17_1n-8t) | 21.2 |  |
| 10-Heptadecenoic acid (C17_1n-7t) | 21.2 |  |
| N-Acetylglutamic acid | 21.3 |  |
| beta-Methylamino-alanine (BMAA) | 21.4 |  |
| trans-Vaccenic acid OR Oleic acid (C18_1n-9c) | 21.4 |  |
| Malic acid peak 1 | 21.7 |  |
| Cysteine | 21.9 |  |
| 10,13-dimethyltetradecanoic acid (C17_0) | 22.0 |  |
| 2-Oxovaleric acid | 22.2 |  |
| Heneicosanoic acid (C21_0) | 22.4 |  |
| Pyruvic acid | 22.6 |  |
| Glutathione | 23.9 |  |
| Palmitelaidic acid (C16_1n-9c) | 23.9 |  |
| Myristoleic acid (C14_1n-5c) | 24.0 |  |
| DL-3-Aminoisobutyric acid | 24.6 |  |
| 16.2858 min (-)-O-Acetylmalic anhydride | 25.0 |  |
| Conjugated linoleic acid (C18_2n-9,11c)OR Linoleic acid (C18_2n-6,9c) OR 10,12-octadecadienoic acid (C18_2n-10,12c) | 25.6 |  |
| Margaric acid (C17_0) | 25.6 |  |
| Ornithine | 25.9 |  |
| Syringic acid | 26.2 |  |
| Cabamic acid | 26.4 |  |
| Malonic acid | 26.4 |  |
| Glutaric acid | 27.4 |  |
| Succinic acid | 27.5 |  |
| Tyrosine | 27.6 |  |
| Suberic acid | 28.5 |  |
| Itaconic acid | 28.8 |  |
| Myristic acid (C14_0) | 29.2 |  |
| Oxaloacetic acid | 29.5 |  |
| Unknown 201(100) 117(89.6) 119(86.2) | 5.1 |  |
| Unknown 091(100) 106(54.7) 105(21.8) | 5.6 |  |
| Unknown 112(100) 84(26.4) 41(9.6) | 6.5 |  |
| Unknown 127(100) 159(49.8) 59(48.8) | 7.4 |  |
| Unknown 119(100) 91(99.3) 120(88.2) | 8.1 |  |
| Unknown 070(100) 182(88.0) 113(70.4) | 8.8 |  |
| Unknown 128(100) 42(11.1) 129(7.7) | 9.1 |  |
| Unknown 115(100) 118(95.4) 86(69.3) | 9.5 |  |
| Unknown 071(100) 57(94.4) 85(72.9) | 11.7 |  |
| Unknown 144(100) 88(51.5) 115(46.4) | 11.8 |  |
| Unknown 115(100) 101(38.0) 55(33.4) | 11.9 |  |
| Unknown 127(100) 143(34.9) 101(32.5) | 12.0 |  |
| Unknown 128(100) 42(0.90) 129(0.88) | 12.2 |  |
| Unknown 129(100) 157(62.8) 97(29.6) | 12.4 |  |
| Unknown 115(100) 100(70.4) 56(60.1) | 13.1 |  |
| Unknown 102(100) 70(69.2) 128(43.5) | 14.1 |  |
| Unknown 074(100) 46(21.3) 59(0.97) | 14.4 |  |
| Unknown 130(100) 70(47.4) 98(19.7) | 14.5 |  |
| Unknown 125(100) 184(90.5) 96(54.3) | 15.7 |  |
| Unknown 126(100) 127(46.7) 59(33.2) | 15.8 |  |
| Unknown 113(100) 85(48.1) 59(21.2) | 16.5 |  |
| Unknown 071(100) 57(92.3) 43(75.3) (split peak 2) | 18.2 |  |
| Unknown 144(100) 88(66.2) 70(62.9) | 18.4 |  |
| Unknown 113(100) 85(48.8) 114(20.4) | 19.0 |  |
| Unknown 128(100) 129(0.71) 42(0.67) | 19.6 |  |
| Unknown 202(100) 158(43.9) 114(24.6) | 20.7 |  |
| Unknown 102(100) 58(0.86) 59(0.75) | 21.4 |  |
| Unknown 249(100) 57(31.8) 264(22.9) | 22.2 |  |
| Unknown 074(100) 87(68.8) 55(33.1) | 22.8 |  |
| Unknown 091(100) 92(23.6) 120(21.2) | 22.9 |  |
| Unknown 128(100) 139(21.1) 42(19.1) | 23.4 |  |
| Unknown 101(100) 119(80.5) 74(35.3) | 23.5 |  |
| Unknown 091(100) 134(81.6) 65(16.0) | 24.6 |  |
| Unknown 166(100) 139(94.5) 111(42.6) | 24.6 |  |
| Unknown 252(100) 296(84.8) 236(47.6) | 24.9 |  |
| Unknown 091(100) 43(80.7) 57(75.1) | 25.9 |  |
| Unknown 074(100) 87(69.0) 43(32.9) | 26.1 |  |

CV: Coefficient of variation


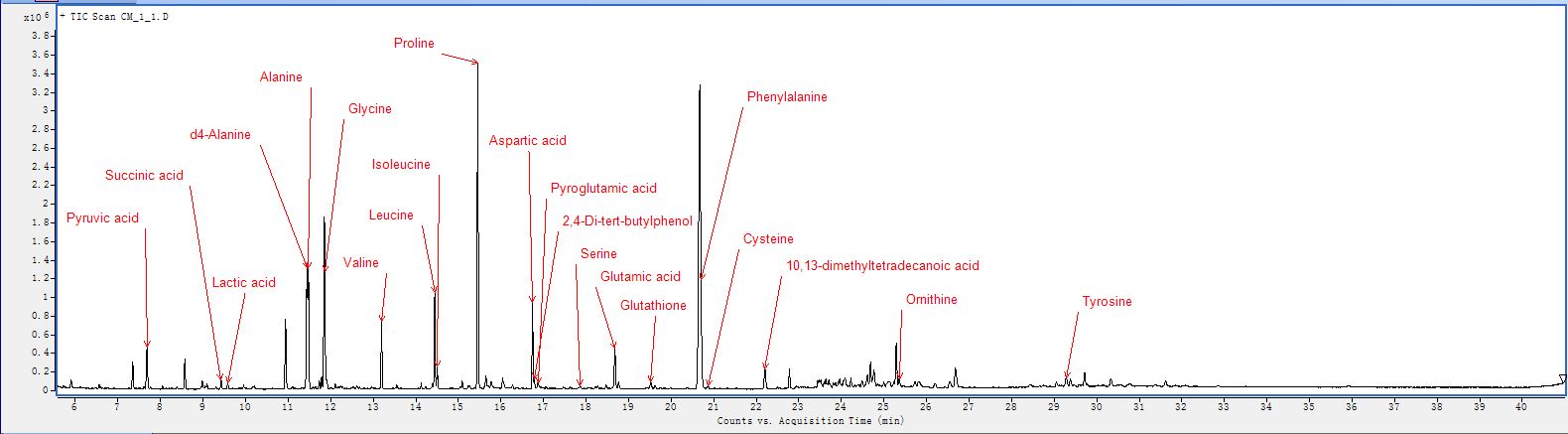
**Supplementary Figure 1. Representative total ion chromatogram (TIC) of ICP hair metabolome.**
